# Assessing healthcare experiences and barriers to care among individuals with ectodermal dysplasia

**DOI:** 10.1186/s13023-026-04247-z

**Published:** 2026-03-05

**Authors:** Amanda K. Swanson, Kimberly J. Hammersmith, Jin Peng, Kaitrin K. Kramer, Andrew Wapner, Beau D. Meyer

**Affiliations:** 1 Nationwide Children’s Hospital 700 Children’s Drive Columbus, Columbus, OH 43210 USA; 2 College of Public Health 250 Cunz Hall 1841 Neil Ave. Columbus, Columbus, OH 43210 USA

## Abstract

**Purpose:**

To conduct a scoping assessment of barriers and facilitators to medical and dental care among individuals and caregivers affected by ectodermal dysplasia (ED).

**Methods:**

A sequential quantitative and qualitative approach was employed using web-based questionnaires and focus groups. A 76-item questionnaire was developed using a combination of validated instruments and expert opinion. Sections included diagnosis, insurance coverage, oral health status, and access to care. Descriptive statistics and bivariate methods were used to compare medical versus dental access to and satisfaction with care. Focus groups were conducted using a semi-structured guide with caregivers of affected children and individuals with ED. Interview recordings were transcribed verbatim, coded, and analyzed inductively using MaxQDA software.

**Results:**

A total of 174 questionnaire responses were received. Access to and satisfaction with dental care was lower than medical care. Subjects reported finances as the largest barrier to dental care. Eight focus groups were conducted. Most frequently cited frustrations included insurance delays or denials, dismissive provider attitudes, and fragmented coordination of care, all of which placed a time-intensive burden on the affected individual or caregiver. Self-advocacy was common and necessary in accessing proper resources. Families valued the sense of community gained from support organizations and interactions with other affected individuals.

**Conclusions:**

Effective providers demonstrate empathy, curiosity, and willingness to collaborate with their patients and other specialists. Families seek providers who demonstrate honesty, active listening, and open-mindedness to learn. Fostering skills of self-advocacy among families and encouraging deliberative patient-provider relationships in healthcare education hold promise for improving rare disease care.

**Supplementary information:**

The online version contains supplementary material available at 10.1186/s13023-026-04247-z.

## Introduction

In the United States, a rare disease is defined as a condition affecting fewer than one in 200,000 people [1] Rare diseases may be uncommon individually, but collectively, they affect approximately 10% of the U.S. population, or about 30 million people [1]. Affected individuals face unique challenges such as diagnostic and treatment uncertainty, constraints of provider expertise, lack of available knowledge about their condition, fragmented specialist care, and extensive onus of care coordination on the affected individual or caregiver [2, 3]. Rare diseases are costly both for the affected individual and the healthcare system, representing a significant public health issue [4]. Each rare disease has its own unique and complex challenges.

Ectodermal dysplasia (ED) is one such example. ED refers to any of a heterogenous collection of hereditary conditions affecting the development and/or homeostasis of two or more ectoderm derivatives, including hair, teeth, nails, and certain glands [5]. Clinical manifestations may include hypohidrosis, hypotrichosis, hypodontia, craniofacial and limb dysmorphology, nail dystrophy, skin atrophy, and in some cases, associated immunodeficiency, feeding problems, and height deficits. Though previous classifications relied on phenotype only, rapid advancements in genetic testing facilitated an updated classification scheme combining molecular pathogenesis and clinical manifestations [5, 6]. Forty-nine ED subtypes with molecularly confirmed etiology are known.[6] No FDA-approved treatments exist, though in-utero gene therapies are being explored [7]. Clinical management is largely symptomatic including oral rehabilitation for missing or misshapen teeth, cooling devices to accommodate for hypohidrosis, surgical correction of facial deformities, speech and feeding therapy, and lifestyle adjustments to maximize health and comfort. The prevalence of ED is not known in the United States, though European registries and population cohort studies estimate X-linked hypohidrotic ED, the most common type, between 1.6 and 21.9 per 100,000 people [8, 9]. Recent pilot studies are using multicenter databases of electronic health records to estimate prevalence in the United States [10].

Researchers are also interested in the patient experience and challenges faced in seeking healthcare. Sizeable care gaps have been identified for the overall rare disease population [11–13], but these studies broadly group all conditions together, which fails to include nuances accompanying an ED diagnosis. Previous ED-specific investigations have demonstrated impaired overall and oral health-related quality of life [14, 15], with particularly high financial implications of necessary oral rehabilitation [16]. Nonetheless, insight into the patient and caregiver experience is largely based on individual anecdotes and case reports [17–19].

The SDOH framework provides a lens for understanding how socioeconomic position and health-system factors influence the lived experiences and care challenges of individuals with rare conditions like ED [20]. It offers valuable insight because barriers experienced by this population arise not simply from the condition’s clinical features, but from structural factors that strongly influence care access and resulting health outcomes. No assessments of social determinants of health (SDOH) and care-seeking challenges in patients with ED have been published. Similar challenges in long-term care coordination and psychosocial burden have been documented in other rare congenital conditions, such as congenital hypothyroidism, where delayed diagnosis and inconsistent multidisciplinary follow-up influence developmental outcomes [21].

The objective of this study was to comprehensively evaluate SDOH, provider satisfaction, and access to care among patients with ED and their families. When possible, secondary aims compared experiences seeking medical care to dental care within the sample, and compared provider satisfaction, oral health status, and SDOH to established norms.

## Methods

This study included sequential quantitative and qualitative components and was approved by Nationwide Children’s Hospital IRB #00004058.

### Part A: Quantitative Component

#### Recruitment

Questionnaire inclusion criteria were 1) any adult (age 18+) with clinically confirmed or self-diagnosed ED, or caregiver of a child (age 0–18) with ED, 2) English literate, and 3) residing in North America. Affected individuals who were also caregivers completed the questionnaire for themselves only. Caregivers with multiple affected children responded for the oldest child only.

The questionnaire was distributed via email to those receiving communications from the National Foundation for ectodermal Dysplasias (NFED), who has approximately 10,700 individuals in their database. A flyer was advertised on NFED social media pages and posted at the NFED family conference in July 2024. It was also shared on the American cleft palate craniofacial association online forum.

Responses were collected from July 2024 to November 2024. The final question asked if the respondent would like to participate in a focus group (Part B), where contact information was collected separately.

#### Instrument Development

A REDCap [22] questionnaire was developed using validated instruments from the literature including the patient satisfaction questionnaire-18 (PSQ-18) [23] and National health and Nutrition Examination Survey (NHANES) [24] published surveys for other rare diseases [12, 13], and expert opinion. The questionnaire was reviewed for relevance and impact by two NFED staff members (laypeople) and five medical providers with experience treating patients with ED (prosthodontist, pediatrician, pediatric dentist, orthodontist, and general dentist). A group of non-healthcare providers pilot tested for readability and timing.

Respondents first indicated whether they were an adult or a caregiver of an ED affected individual. The instrument then branched into two question sets with tailored language, but asked the same questions. The questionnaire included 76 questions subdivided into four domains: diagnosis, insurance coverage, oral health status, and access to and satisfaction with care. Demographic, socioeconomic, and ED subtype data were also collected. A copy of the questionnaire is included (Additional file 1).

#### Analysis

All analyses were performed using R version 4.4.2 (R Foundation for Statistical Computing, Vienna, Austria). Summary statistics were calculated to describe respondent demographics.

While questionnaire data from affected individuals and caregivers was collected separately, many of the caregivers of a child with ED were also affected with ED themselves, as expected for a genetic condition. Thus, the decision was made to examine the results combined.

Analysis was stratified by medical versus dental care, then compared using Chi-squared tests. Travel distances for care were compared using Wilcoxon Signed-Rank Tests because the data were not normally distributed and were highly skewed. Mean and standard deviation for PSQ-18 outcome measures were calculated and compared to other rare disease reference data using two sample t-tests. Internal reliability for the PSQ-18 subscales was assessed using Cronbach’s alpha. Identical adult and caregiver items were combined, and negatively worded items were reverse coded so that higher scores represented greater satisfaction. Cronbach’s alpha values are reported for each subscale.

Frequencies and percentages of NHANES dental questions were calculated and compared with the general population using Chi-squared tests. Stratified analyses for sociodemographic variables were conducted to predict outcomes of adequacy of financial, medical, and dental support using Fisher’s exact test. Because the number of events was limited, stratified analyses rather than multivariable modeling were used. *p* < 0.05 was considered statistically significant.

### Part B: Qualitative Component

#### Recruitment

Part A respondents indicating interest in the qualitative interviews were recruited for Part B via email, which was collected separately following submission of the anonymous survey. All respondents who expressed interest were given equal opportunity to participate. Additional inclusion criteria included technology needed to participate via zoom workplace (version 6.2.11). Subjects were enrolled continuously from July 2024 to November 2024 and given a choice of dates. Participation was voluntary and uncompensated.

#### Instrument development

A semi-structured interview guide was developed containing a facilitator script and open-ended inquiries aligning with Part a topics (additional file 2). Questions explored diagnostic experiences, provider satisfaction, oral health perspectives, and overall access to care. Focus groups continued until thematic saturation was reached. Participants received the interview guide two days in advance for optional review

#### Interviews

The primary author (A.S.) facilitated in-depth virtual interviews in October and November 2024. Each group contained 2–5 participants, lasted one hour, and included a mix of ED-affected individuals and caregivers. Consent was obtained at the beginning of each session. Participants shared information to the extent that they felt comfortable and could terminate participation at any time. The facilitator used the interview guide plus additional probing questions as the conversation evolved. Each session was recorded for transcription.

#### Analysis

Recordings were transcribed using MAXQDA24 (VERBI software, Berlin, Germany) and cross-checked with the audio for accuracy, with identifying details removed.

Transcripts were coded inductively using qualitative descriptions and thematic analysis. A common codebook was developed from the interview guide and discussion notes. Two researchers coded the first two transcripts (A.S., B.M.) and iteratively refined the codebook before applying to the remaining sessions. Emerging themes were distilled from coded segments and paired with narrative summaries and illustrative quotes for each.

## Results

### Part A: Quantitative Component

Of 174 questionnaires initiated, 111 contained complete responses. Demographics were summarized in Table 1. Most respondents (*n* = 108, 62.1%) were caregivers of a child with ED. Participants were mostly female, Caucasian, with a bachelor’s or graduate degree, and annual household income above $100,000. Average respondent age was 47 (range 21 to 80), with hypohidrotic ED as the most common subtype (49%). Over half had a confirmed genetic diagnosis (51.8% of affected adults, 74% of children). Most had private medical and dental insurance.

**Table 1:** A. Demographics of survey respondents

	Caregiver of child	Affected adult	Combined
**Survey respondents**	n = 108, (62.1%)	n = 66, (37.9%)	n = 174 (100%)
**Gender:** Female Male	[affected child] 34 (34.7) 64 (65.3)	N/A	141 (86) 23 (14)
**Race:** American Indian/AN Asian Black/AA Hispanic or Latino/a Middle Eastern Native Hawaiian/PI White or Caucasian Other	2 (3) 1 (1.5) 2 (3) 2 (3) 0 (0) 0 (0) 58 (87.9) 1 (1.5)	1 (1) 4 (3.8) 4 (3.8) 7 (6.7) 0 (0) 0 (0) 87 (83.7) 1 (1)	3 (1.8) 5 (2.9) 6 (3.5) 9 (5.3) 0 (0) 0 (0) 145 (85.2) 2 (1.2)
**Education:** Some high school High school diploma Associates/technical Bachelor’s degree Graduate degree	N/A	N/A	3 (1.8) 29 (17.6) 29 (17.6) 51 (30.9) 53 (32.1)
**Annual income:** < 25K 25–49,999 50–74,999 75–99,999 > 100K Rather not say	N/A	N/A	9 (5.5) 12 (7.3) 17 (10.4) 26 (15.9) 77 (47) 23 (14)
**Geography:** Large city ( > 50K) Medium city (5-50K) Small town/city ( < 5K)	N/A	N/A	71 (44.1) 58 (36) 32 (19.9)
**B.** Demographics of ED-affected individuals.			
**Age** (mean, range)	13 (0–47)	47 (21–80)	N/A
**Type of ED:** HED HED w/immune def EEC AEC Clouston Goltz IP Other Unknown	47 (49) 5 (5.2) 3 (3.1) 4 (4.2) 0 (0) 6 (6.2) 4 (4.2) 13 (13.5) 14 (14.6)	28 (49.1) 2 (3.5) 5 (8.8) 1 (1.8) 3 (5.3) 3 (5.3) 1 (1.8) 6 (10.5) 8 (14)	75 (49) 7 (4.6) 8 (5.2) 5 (3.3) 3 (2) 9 (5.9) 5 (3.3) 19 (12.4) 22 (14.4)
**Confirmed genetic diagnosis:** Yes No	71 (39.7) 24 (25)	26 (46.4) 29 (51.8)	97 (64.7) 53 (35.3)
**Number of people in family with ED** (mean, range)	N/A	N/A	1.8 (0–10)
**Medical insurance:** Private – employer Private – purchased Medicare Medicaid Military healthcare Other None Not sure	70 (65.4) 1 (0.9) 2 (1.9) 19 (17.8) 5 (46.7) 8 (74.8) 1 (0.9) 1 (0.9)	34 (54.8) 4 (6.5) 11 (17.7) 6 (9.7) 0 (0) 5 (8.1) 2 (3.2) 0 (0)	104 (61.5) 5 (3) 13 (7.7) 25 (14.8) 5 (3) 13 (7.7) 3 (1.8) 1 (0.6)
**Dental insurance:** Private Public None Not sure	72 (70.6) 20 (19.6) 6 (5.9) 4 (3.9)	35 (63.6) 2 (3.6) 15 (27.3) 3 (5.5)	107 (68.2) 22 (14) 21 (13.4) 7 (4.5)

Participants’ views on access to dental versus medical care were compared in Table 2. Finances significantly limited access to dental versus medical care (*p* = 0.03). No other access measures differed between the two.

**Table 2:** Comparing differences in access to medical and dental care

Outcome	MEDICAL (*n* = 174, 100%)	DENTAL (*n* = 174, 100%)	p-value
Did any of the following limit your ability to get medical or dental care for your ectodermal dysplasia?			
Finances	AA: 15 (11.4) M: 17 (12.9) AF: 10 (7.58) S: 36 (27.3) N: 54 (40.9)	AA: 31 (24.8) M: 21 (16.8) AF: 8 (6.4) S: 29 (23.2) N: 36 (28.8)	0.04
Travel distance	AA: 7 (5.4) M: 8 (6.2) AF: 6 (4.7) S: 37 (28.7) N: 71 (55)	AA: 11 (9.1) M: 6 (5) AF: 10 (8.3) S: 31 (25.6) N: 63 (52.1)	0.57
Difficulty getting time of school/work	AA: 8 (6.5) M: 9 (7.3) AF: 10 (8.1) S: 35 (28.2) N: 62 (50)	AA: 9 (7.6) M: 5 (4.2) AF: 13 (11) S: 34 (28.8) N: 57 (48.3)	0.80
Lack of childcare	AA: 2 (1.9) M: 3 (2.9) AF: 5 (4.8) S: 18 (17.1) N: 74 (70.5)	AA: 4 (4) M: 2 (2) AF: 2 (2) S: 14 (14.1) N: 77 (77.8)	0.62
Lack of or delay in referrals	AA: 10 (8.5) M: 5 (4.3) AF: 15 (12.8) S: 44 (37.6) N: 43 (36.8)	AA: 12 (10.4) M: 4 (3.5) AF: 15 (13) S: 33 (28.7) N: 51 (44.3)	0.64
Challenges with insurance coverage	AA: 35 (27.3) M: 19 (14.8) AF: 12 (9.4) S: 28 (21.9) N: 34 (26.6)	AA: 50 (40.7) M: 16 (13) AF: 10 (8.1) S: 16 (13) N: 31 (25.2)	0.17
How far do you usually travel (in miles) to get medical or dental care? (mean, sd OR median, IQR)	Mean = 53.9, sd = 67.8	Mean = 61.8, sd = 82.3	0.99
What is the most distance you have traveled (in miles) to get medical or dental care? (mean, sd or median, IQR)	Mean = 114.2, sd = 101.2	Mean = 110.6, sd = 102.9	0.52
The first medical or dental provider I saw when I started having problems with ectodermal dysplasia was:			
Knowledgeable of ED	P: 66 (48.9) F: 26 (19.3) N: 11 (8.1) G: 20 (14.8) E: 12 (8.9)	P: 56 (42.7) F: 16 (12.2) N: 9 (6.9) G: 32 (24.4) E: 18 (13.7)	0.12
Willing to ask other colleagues for help	P: 33 (25.4) F: 19 (14.6) N: 17 (13.1) G: 29 (22.3) E: 32 (24.6)	P: 39 (31.2) F: 17 (13.6) N: 13 (10.4) G: 21 (16.8) E: 35 (28)	0.65
Willing to research ED themselves	P: 37 (28.5) F: 20 (15.4) N: 17 (13.1) G: 27 (20.8) E: 29 (22.3)	P: 42 (33.9) F: 15 (12.1) N: 14 (11.3) G: 24 (19.4) E: 29 (23.4)	0.85

AA = Almost always *p* = poor *M* = Most of the time F = fair AF = About half of the time *N* = neutral S = Sometimes G = good *N* = Never E = excellent *P<0.05; Changes in self-efficacy pre- and post-module review among the test group only were measured using Wilcoxon matched-pairs signed-rank tests

Table 3 summarized provider satisfaction (PSQ-18) among study participants and included general population and rare disease reference groups [13, 23]. Outcome measures include general satisfaction, technical quality, finances, time spent with provider, communication, accessibility, and provider interpersonal manner. All outcomes were significantly lower among study participants compared to the general population. Compared to the rare disease group, only financial satisfaction differed significantly. The PSQ-18 subscales demonstrated acceptable internal reliability, shown by Cronbach’s alpha coefficients of 0.85 for general satisfaction, 0.78 for technical quality, 0.77 for financial aspects, 0.80 for time spent with provider, 0.75 for communication, 0.65 for accessibility and convenience, and 0.69 for interpersonal manner.

**Table 3:** External validation of the PSQ-18 for individuals with ectodermal dysplasia

Outcome (mean, SD)	Patients with rare disease* (*n* = 1128)	Reference data** (*n* = 2,197)	Current sample (*n* = 174)	p-value (rare disease vs. current sample)	p-value (reference vs. current sample)
General satisfaction	3.09 (1.19)	3.58 (0.94)	2.93 (1.19)	0.10	< 0.001
Technical quality	3.39 (1.04)	3.68 (0.76)	3.24 (1.25)	0.13	< 0.001
Financial	3.25 (1.18)	3.78 (0.94)	2.87 (1.34)	> 0.001	< 0.001
Time spent with healthcare provider	3.42 (1.12)	3.59 (0.94)	3.34 (1.12)	0.38	0.005
Communication	3.47 (1.06)	3.74 (0.87)	3.32 (1.20)	0.12	< 0.001
Accessibility	3.29 (1.00)	3.76 (0.74)	3.16 (1.19)	0.17	< 0.001
Provider interpersonal manner	3.90 (0.93)	4.09 (0.69)	3.82 (1.03)	0.34	< 0.001

*Bogart et al. [13] **Marshall et al. [23] ***p < 0.05; Calculated using two sample independent t-tests

Table 4 , Table 5, and Table 6 summarized remaining results. ED-affected individuals had significantly different responses than the general population across all dental questions (Table 4). Compared to other rare diseases [13], ED-affected individuals reported similar medical support, but different dental, financial, psychological, and social support (Table 5). When sociodemographic variables were analyzed for participants’ perceived levels of support, numerous significant associations were found (Table 6). Lack of financial support was significantly associated with race, with 88% of Black participants reporting financial barriers compared to 49% of White respondents. Stratified analyses found no significant associations between perceived medical support and all sociodemographic variables tested (race, education, income, geography and gender). Analyses did, however, reveal a significant association between household income and lack of dental support. Across all subgroups, approximately one in four participants reported lacking dental support.

**Table 4:** External validation of NHANES dental questions

Outcome	Current sample of ED-affected individuals (*n* = 174, 100%)	General population*	p-value
Overall, how would you rate the health of your teeth and gums?	E: 16 V: 27 G: 39 F: 31 P: 29	E: 2634 V: 2950 G: 3539 F: 1656 P: 944	< 0.001
In the last year, how often have you felt life in general was less satisfying because of problems with your teeth, mouth, or dentures?	VO: 26 FO: 19 O: 45 HE: 23 N: 26	VO: 214 FO: 250 O: 685 HE: 1389 N: 5254	< 0.001
In the last year, how often have you had difficulty doing usual jobs or attending school because of problems with your teeth, mouth or dentures?	VO: 10 FO: 13 O: 30 HE: 34 N: 52	VO: 64 FO: 92 O: 227 HE: 671 N: 6738	< 0.001
In the last year, how often have you avoided particular foods because of problems with your teeth, mouth, or dentures?	VO: 44 FO: 26 O: 36 HE: 15 N: 19	VO: 361 FO: 300 O: 862 HE: 1319 N: 4953	< 0.001
In the last year, how often have you been self-conscious or embarrassed because of your teeth, mouth, or dentures?	VO: 35 FO: 21 O: 36 HE: 15 N: 32	VO: 414 FO: 279 O: 646 HE: 1025 N: 5436	< 0.001

E = Excellent VO = Very often V = Very good FO = Fairly often G = Good O = Occasionally *p* = Poor HE = Hardly ever F = Fair *N* = Never *National Health and Nutrition Examination Survey [24]

**Table 5:** Comparing differences in support received by patients affected by ectodermal dysplasia

Outcome	Patients with ectodermal dysplasia (*n* = 174, 100%)	Patients with rare disease* (*n* = 905, 100%)	p-value
Do you agree or disagree that you have received sufficient support in the following areas?			
Medical (i.e. doctors, nurses)	SA: 33 A: 41 N: 28 D: 20 SD: 16	SA: 233 A: 308 N: 131 D: 132 SD: 101	0.48
Dental	SA: 38 A: 37 N: 16 D: 21 SD: 21	SA: 58 A: 130 N: 185 D: 53 SD: 55	< 0.001
Social (i.e. family, friends, church members)	SA: 55 A: 36 N: 19 D: 15 SD: 10	SA: 168 A: 247 N: 193 D: 154 SD: 87	< 0.001
Financial (i.e. insurance coverage)	SA: 7 A: 23 N: 18 D: 34 SD: 51	SA: 114 A: 274 N: 216 D: 124 SD: 120	< 0.001
Psychological (i.e. mental health care, counselors)	SA: 10 A: 20 N: 43 D: 16 SD: 23	SA: 59 A: 136 N: 215 D: 145 SD: 76	0.047
How long after first seeking medical help did it take to get a confirmed diagnosis? (mean, SD)	2.21 (3.10) in years	3.24 (2.01) in years	< 0.001
How many providers have you seen to get a diagnosis? (mean, SD)	3.06 (3.68)	4.50 (2.90)	< 0.001
Looking back, were you given enough information on you/your child’s condition? Yes No Don’t know/NA	Yes: 51 No: 77 Don’t know/NA: 46	Yes: 443 No: 434	0.03

SA = Strongly agree A = Agree *N* = Neutral D = Disagree SD = Strongly disagree *Bogart et al.[13]

**Table 6:** Stratified analysis of sociodemographic factors associated with lack of financial, medical, and dental support

Sociodemographic Factor	Category	Total N	% Reporting lack of financial support (n)	*p***-value**	% Reporting lack of medical support (n)	*p***-value**	% Reporting lack of dental support (n)	*p***-value**
Race	White	149	49.0 (73)	0.02	22.8 (34)	0.87	24.2 (36)	0.26
	Black	6	83.3 (5)		16.7 (1)		50.0 (3)	
	Other	8	87.5 (7)		12.5 (1)		37.5 (3)	
Education	Some high school or less	3	33.3 (1)	0.96	33.3 (1)	0.76	33.3 (1)	0.43
	High school diploma or GED	29	44.8 (13)		24.1 (7)		27.6 (8)	
	Associates or technical degree	29	55.2 (16)		24.1 (7)		37.9 (11)	
	Bachelor’s degree	51	51.0 (26)		19.6 (10)		19.6 (10)	
	Graduate degree	53	54.7 (29)		20.8 (11)		22.6 (12)	
Household income (USD, 2023)	Under $25,000	9	55.6 (5)	0.9	44.4 (4)	0.12	44.4 (4)	0.001
	$25,000– 49,999	12	41.7 (5)		16.7 (2)		8.3 (1)	
	$50,000– 74,999	17	47.1 (8)		35.3 (6)		64.7 (11)	
	$75,000– 99,999	26	53.8 (14)		19.2 (5)		15.4 (4)	
	Over $100,000	77	50.6 (39)		20.8 (16)		22.1 (17)	
	Rather not say	23	60.9 (14)		13.0 (3)		21.7 (5)	
Geography	Large city ( > 50,000 residents)	71	54.9 (39)	0.87	15.5 (11)	0.14	22.5 (16)	0.41
	Medium city (5,000– 50,000 residents)	58	50.0 (29)		27.6 (16)		29.3 (17)	
	Small town or city ( < 5,000 residents)	32	53.1 (17)		28.1 (9)		28.1 (9)	
Gender	Female	141	52.5 (74)	0.38	23.4 (33)	0.76	28.4 (40)	0.14
	Male	23	47.8 (11)		13.0 (3)		8.7 (2)	

### Part B: Qualitative Component

Eight focus groups were completed with 23 individuals, leading to the identification of seven key themes which were summarized alongside illustrative quotes in Table 7.

**Table 7:** Domains, themes, and representative quotations from qualitative interviews

Domain	Theme	Quotation
Genetic diagnosis	Reassurance, family planning, school accommodations, specialist access	“With my daughter […] she follows a very similar pattern to other children with this diagnosis, and I feel reassured that we’re providing her the best care because we have a better idea of what’s going on.”
Provider Interactions	Positive: open-minded, creative, questioning Negative: dismissive, rigid, discontinuous	“I love that I have doctors that have their eyes and ears open for new things, because my kid is not a one size fits all situation.” “If you see or hear a condition you are not familiar with, listen to the patient more because you may not be familiar with it, but if we’ve been living with it, we can teach you.”
Care Coordination	Burden on caregivers, poor system integration, geographic barriers	“Sometimes I feel like I have a part time job […] like I’m phoning the ENT office, who then may phone the pediatrician here. And then it’s like, oh, well, you’ve got to send a fax. And I’m like, nobody sends faxes. Can you do an email? And then three of her doctors are in the same building at the same hospital, but I have to send the same report separately to each of them.” “If we have to travel to get this done, we will. I was like, well, I was only four or five hours away. And so thankfully, we have the resources and the motivation to do that.”
Mental Health	Emotional burden, parental concern, school-related challenges	“I think that’s been a huge part of it, though, is finding your community and trying to kind of figure out, how can I help my kids find theirs because I worry about the same things. I worry that my kids look different and they get asked quite often, and it’s hard.”
Oral Health Attitudes	Functional priority, self-confidence, value	“So now he just got the best mouthpiece. It cost me $12,000, but the best piece he’s ever had, because he has the implants in now. And I see his confidence is so different than what it was ten years ago. Like I’ve never seen him smile. And so now he like beams ear to ear in pictures. I’m like, oh my God, what a difference it makes.” “When you don’t have a single lower tooth, his ability to eat, his ability to speak, these are two things you need to communicate with the world and to stay healthy. If that’s not high priority level, I don’t know what is. Because if you can’t eat, you can’t survive. If you can’t speak, you can’t communicate with the world around you.”
Advocacy	Constant need to advocate in all environments, research participation	“Once our kid leaves the house, […] we have to advocate for him at school. We have to advocate for him at social events or sporting activities and things like that […]. His eyes are more sensitive because of this. His teeth are a little bit different because of this. He talks a little bit differently because of this, you know, and so I think, the advocacy is kind of a train 24 hours a day, seven days a week, 365 days a year on some level.” “The pediatric dermatologist loves to always show everyone, the residents and fellows who are there when my son comes in. I get it, I want learning, but they all come in and then they want to take pictures […] so I think that gets a little bit sensitive in a way where, I want to educate and I want people to see and I’m happy to answer questions, but we’re not going to throw them up on a PowerPoint slide.”
Community & Resilience	Value of shared experience and connection	“The biggest thing for me has been finding community and I’ve really enjoyed you ladies. I’ve gotten so much from you all today. So thank you for the opportunity to participate, because I think, at first I was like, oh my gosh, the odds are like one in 15,000. But now we’re finding others and hearing stories. I found a lot of value in the Facebook page as well, specifically for questions or comments or read stories and experiences.”

Participants had mixed views on the utility of a genetic diagnosis. “Good to know, but it didn’t change anything” was a common sentiment. The most common benefit was for reassurance, closely followed by family planning. Some mentioned that it prompted providers to take their concerns seriously, facilitating access to specialty care. Others mentioned improved school accommodations for their child (i.e. 504/IEP).

Positive provider experiences were defined by active listening, collaboration, and curiosity. Families valued providers’ creativity and honesty about knowledge gaps. Negative experiences involved dismissiveness, overconfidence, or rigidity. Families were more troubled by rigidity and close-mindedness than they were by someone’s lack of knowledge about ED, so long as they were willing to ask questions and eager to learn. Continuity of care emerged as particularly important, with families expressing fatigue from repeatedly explaining their condition to new providers.

Families consistently described the burden of managing care coordination themselves – navigating referrals, locating providers, appealing insurance, and managing inter-specialist communication. Those with access to multidisciplinary teams (i.e. academic centers, craniofacial clinics) reported better experiences, though not always accessible to those in rural areas. Many cited the nuisance of traveling but it did not prevent them from seeking care. Medical insurance was helpful, albeit tedious and time-consuming, but often hindered timely access to treatment which was particularly troubling for growing children.

The psychosocial impact of ED varied. Caregivers shared negative feelings of frustration, anxiety, and loneliness, balanced by positive emotions of resilience and hope. Bullying and fear of social exclusion were common concerns, particularly in school settings that lacked awareness about ED.

Oral health concerns were prominent, with participants expressing frustration over treatment delays and inconsistent provider experiences. Actionable care within the pediatric population was appreciated, and while esthetics were noted as important, families more quickly cited functional benefits like feeding and speech. Though participants were not opposed to less experienced providers, they found value in offices with ED-specific expertise, often traveling far to reach them. Many expressed a fatalistic view about dental insurance and found it prohibitive to achieving timely care.

Self-advocacy was not explicitly queried but emerged consistently. Individuals cited the importance of advocating for themselves during medical encounters, with caregivers noting additional difficulty empowering their children to be their own advocates when transitioning from pediatric to adult care. Participants voiced a strong desire for research and willingness to contribute but also noted discomfort when feeling put on display for learners because of their differences.

Lastly, community played a vital role. Participants relied on NFED resources, social media groups, and family conferences to find connection, information, and support. They expressed appreciation for the shared learning and camaraderie during the focus groups themselves, with many expressing desire to stay connected afterwards.

## Discussion

### Study Value

Both the quantitative instrument and the qualitative focus groups offered insight on care-seeking experiences. The focus groups procured deeper insight into the quantitative responses, generating additional ideas to improve the care experience not previously identified by researchers.

This study contributes an understanding of effective catalysts of care for patients with ED and their families. The supportive sense of community from individuals and advocacy organizations mirrors what has been reported generally for rare diseases, particularly regarding the critical role of support groups and key themes of loneliness, calling for greater psychosocial assistance and informal peer support [2, 3, 11, 25].

The results confirmed barriers to care for patients with ED and their families, most notably difficulty finding a provider, challenges with insurance, and fragmented specialist communication. Other studies reported similar barriers, including finances, time, burden of care coordination, and lack of professional guidance [3, 13]. This study also confirmed that ED-affected individuals experience significantly different dental challenges compared to the broader population [24].

The emergence of certain barriers for individuals in the study can be explained by interpreting these results within existing conceptual frameworks for social determinants of oral health inequalities. A model by Daly et al. explains the complex interrelationships between structural determinants and intermediary determinants of oral health, which can be applied to individuals with rare diseases like ED [20]. During the focus groups, study participants mentioned structural barriers around social and welfare policies, which also coincided with questionnaire responses. Socioeconomic position, including gender, race, geography, education, and income were all considered. Intermediary barriers faced by participants centered on health services as the key measure in this study and the primary outcome examined. This model further explains how these barriers interact by demonstrating the influence on oral health inequalities faced by those with ED compared to the general population. It also contextualizes how the social gradient differs for people with rare diseases like ED beyond the biological processes at the individual level [20].

Stratified analyses revealed a racial disparity in financial support, with non-White participants more likely to experience financial barriers than White participants. However, rates did not differ by income, education, gender, or geography, suggesting that the financial burden associated with ectodermal dysplasia care is pervasive across socioeconomic strata.

Income was the only sociodemographic factor significantly associated with perceived lack of *dental* support, with lower-income participants reporting greater difficulty accessing or affording dental care. These results align with national data showing that dental coverage is less comprehensive than medical coverage and that out-of-pocket costs are a major barrier to oral health care, particularly for rare disease populations [14, 16]. Perceived *medical* support did not vary by sociodemographic groupings, which aligns with qualitative themes in Part B highlighting insurance coverage gaps and stronger access to medical specialists compared with dental care.

### Comparison to Literature

Other researchers using comparable methodology found a lack of professional guidance and difficulty streamlining collaboration as key frustrations faced by rare disease caregivers [3]. They reported that diagnosis often influenced the ability to access services [3]. In contrast, the current study’s data found that 81.8% of respondents felt that having a diagnosis did not change their ability to get care for their ED, and the focus groups overwhelmingly concurred. Perhaps these findings differ due to ED care being mostly symptomatic rather than curative, or because ED-specific barriers persist irrespective of diagnostic status. Such differences highlight the importance of tailored approaches to addressing barriers in rare disease care, as challenges may vary significantly by condition. Individuals with rare diseases in other countries have also reported generalized dissatisfaction regarding the level of support provided by their healthcare systems [26].

The current study utilized previous questions and data from other research teams, including one that used the PSQ-18 in a rare disease population [12, 13]. Our data further validated previous research in all areas except financial satisfaction. PSQ-18 results from this study differed significantly from the general population in all seven domains [23].

An individual’s healthcare experiences are often more complicated than can be captured in a simple survey question. For instance, many respondents indicated that travel distance impacted accessing care (65.4% for dental, 38.6% for medical). Focus group participants noted that geography was a complicating factor, but not necessarily one that precluded families from receiving care. From this, we conclude that an access issue exists, though not one that affected individuals are unwilling to overcome. This concurs with previous findings that nearly half of study participants reported traveling 60 miles or more for their rare disease care [13]. Telehealth is one promising strategy to alleviate the travel burden, especially for consultation visits [27, 28].

### Provider Improvements

Tsitsani et al. purported that the most common barriers were at the healthcare system level [2]. Systems-level change is impactful, but can be slow and difficult to achieve. Here, we propose that meaningful action can also be taken at the provider level to facilitate better care for those with ED.

Affected individuals reported willingness to participate in research, consistent with previous findings [12]. However, the way that providers approach interactions at the crossroads of research and clinical care is paramount. Participants were eager to participate, but disliked being seen as a teaching opportunity or put on display for their differences. This presents a dilemma for researchers and academic centers. Providers should emphasize the importance of treating patients as people first, even when education or research is involved.

While it was clear that providers did not always have answers, participants consistently expressed that they were looking for partners in their care. With limited time in the medical training curriculum and evolving best practices, teaching students comprehensively about every rare disease is impractical. Rather, one can learn the mindset necessary to approach these interactions and how to seek necessary information. Encouraging this mindset will allow providers to skillfully treat patients with all rare conditions.

Families most appreciated providers who fostered partnerships and saw patients as the experts. Focus groups substantiated a more complex doctor-patient relationship with ED, which most closely mirrors the mutual participation model [29]. Other studies described how behaviors of rare disease patients influence communication [30]. Interactions are largely patient-directed due to clinicians’ insufficient expertise, which can cause role uncertainty. Empowering patients to assert active involvement in their care may require a shift away from longstanding, more passive roles [31].

### Healthcare System Improvements

Previous work explored the burden of rare diseases and compared the policy landscape across 11 countries, though the U.S. was excluded due to lacking a national rare disease plan and having significant intra-state variation. [32]. Results showed that diagnosis takes a long time and limited expertise exists, calling for improved collaboration and research [32]. Despite coordination challenges, national registries were noted as critical to understand prevalence and treatment trends. [32] Future studies could examine policy feasibility and measure outcomes of formal care coordination and financial assistance as potential strategies to improve care at the systems level.

This study found financial concerns posed a marked challenge, though notably different for medical and dental care, and for ED versus other conditions. As with previous studies, high treatment costs for ED stem from oral problems [16]. In a review of rare diseases, financial challenges were described as a global public health priority, noting a critical need to ensure access to universal, equitable, affordable, and high-quality care [27]. Insurance coverage for medically necessary services resulting from congenital anomalies, including oral rehabilitation, is limited in the U.S., but remains a current advocacy priority [33].

Participants frequently cited difficulty coordinating their own care. Other studies confirmed similar challenges of fragmented care for rare disease patients [2, 3, 32]. Assigning care coordinators through an insurer or accountable care organization may address barriers and reduce caregiver burden. Another avenue is bolstering support from organizations such as the NFED. Less than one-third of study participants reported adequate social support.

### Strengths, Limitations, and Future Work

This study has several strengths. This study included sequential quantitative and qualitative components offering comprehensive insight into the patient and caregiver experience through qualitative and quantitative approaches. The questionnaire was developed specifically for ED utilizing validated and previously published items. Data captured a broad range of ages (0–80 years). The recruitment mechanism and virtual focus groups enriched the geographic diversity.

Regarding limitations, this was a cross-sectional, observational, and largely descriptive study. The North American study population may not be representative worldwide, particularly in the context of other healthcare systems. Similar to other studies, most participants were female [12, 13]. However, most individuals affected by ED, particularly X-linked HED, are male [9]. Recruitment bias is possible as participation was voluntary. The sample was derived almost entirely from NFED channels, which introduces selection bias, and in this case the results may overrepresent individuals with high social resource access, health literacy, and engagement. Thus, the study may have failed to capture those who engage less regularly with resources. The true population of ED patients in North America is unknown, so it is unclear if sample demographics are representative.

Future studies should examine patient empowerment and improving the patient-provider relationship, especially with the patient as the expert. System-level advocacy is needed to improve access and quality of life. Other studies have validated the importance of disease-specific evaluation systems which represents another area of future expansion for individuals affected by ED [34]. Future studies should also examine ED prevalence and explore international populations.

## Conclusions

Understanding the patient experience for individuals and families affected by rare diseases is complex. This study helps clinicians and researchers understand effective facilitators and barriers to care for patients with ED.

- Effective providers caring for patients with ED demonstrate empathy, curiosity, and willingness to collaborate with both patients and other specialists as part of the overall care team.
- Families affected by ED seek providers who demonstrate honesty, eagerness to listen, and an open-minded approach to learn about the specifics of their condition.
- Fostering skills of self-advocacy among families and encouraging deliberative patient-provider relationships in medical education holds promise for improving ED-related care.

## Electronic supplementary material

Below is the link to the electronic supplementary material.

Supplementary Material 1

Supplementary Material 2

## Data Availability

The datasets during and/or analyzed during the current study are available from the corresponding author on reasonable request.
