# Supplementary material for: Assessing healthcare experiences and barriers to care among individuals with ectodermal dysplasia
Source: Orphanet J Rare Dis. 2026 Mar 5;21:147. doi: 10.1186/s13023-026-04247-z (PMC13072512; doi:10.1186/s13023-026-04247-z)
Supplement: Supplementary file 1 — Supplementary Material 1 [file 13023_2026_4247_MOESM1_ESM.pdf]

Assessing healthcare experiences and access to care among individuals with ectodermal dysplasia

CONSENT TO PARTICIPATE IN A CLINICAL RESEARCH STUDY

STUDY TITLE: Assessing healthcare experiences and access to care among individuals with ectodermal dysplasia

PRINCIPAL INVESTIGATOR: Dr. Kimberly Hammersmith, DDS, MPH, MS

CONTACT TELEPHONE NUMBER: 614-722-1651

1)   INTRODUCTION

We invite you to be in this research study. Using this form as a guide, we will explain the study to you. If you have any questions about the study, please ask. If you do not want to be in this study, all regular and standard medical care will still be available to you at Nationwide Children's Hospital. Participation is voluntary. You can leave this study at any time. You will be given a copy of this form.

2)   WHERE WILL THE STUDY BE DONE AND HOW MANY SUBJECTS WILL TAKE PART?

This study will be done at Nationwide Children's Hospital and we hope to enroll 430 participants aged 18 years and older.

3)   WHAT WILL HAPPEN DURING THE STUDY AND HOW LONG WILL IT LAST?

The purpose of this study is to conduct a qualitative assessment on social determinants of health and access to care among patients with ectodermal dysplasia and their families.

Study participation: Subjects will complete one questionnaire at the time of enrollment. Subjects will have the option to participate in a one-time focus group that will last one hour.

4)   WHAT ARE THE RISKS OF BEING IN THIS STUDY?

The risks for this study are no more than what happens in everyday life.

5)   ARE THERE BENEFITS TO TAKING PART IN THIS STUDY?

Although there will be no benefit to you from being in this study, we hope to learn something that could help others.

6)   WHAT ARE THE COSTS AND REIMBURSEMENTS?

You will not be paid to participate in this study.

7)   WHAT HAPPENS IF I DO NOT FINISH THIS STUDY?

It is your choice to be in this study. You may decide to stop being in this study at any time. If you stop being in the study, there will be no penalty or loss of benefits to which you are otherwise entitled. If at any time the Principal Investigator believes that this study is not good for you, the study team will contact you about stopping. If the study instructions are not followed, participation in the study may also be stopped.

8) OTHER IMPORTANT INFORMATION

If you are an employee of Nationwide Children's Hospital or the Research Institute at Nationwide Children's Hospital, your job or performance appraisal will not be affected in any way if you decline to participate or withdraw your consent to participate in this study.:

Nationwide Children's Hospital is a teaching hospital and we are committed to doing research. Doing research will enable us to learn and provide the best care for our patients and families. You may be asked to participate in other research studies in the future. You have the right to decide to participate or decline to participate in any future studies. We will not share your contact information with researchers outside Nationwide Children's Hospital.

9) HOW WILL MY STUDY INFORMATION BE KEPT PRIVATE?

Information collected for this study includes information that can identify you. This is called "protected health information" or PHI. By agreeing to be in this study, you are giving permission to your health care provider to use or disclose (release) your health information that identifies you for the research study described in this form. Information collected is the property of Nationwide Children's Hospital, its affiliated entities, and/or the sponsor.

· PHI that may be used or disclosed will include: E-mail Address

People or Companies authorized to use, disclose, and receive PHI collected or created by this research study:

· PI and study staff

· The Nationwide Children's Hospital Institutional Review Board (the committee that reviews all human subject research)

· Nationwide Children's Hospital internal auditors

Because of the need to give information to these people, absolute confidentiality cannot be guaranteed. Information given to these people may be further disclosed by them and no longer be protected by federal privacy rules.

Reason(s) why the use or disclosure is being made: To contact you in the future. You may decide not to authorize the use and disclosure of your PHI. However, if it is needed for this study, you will not be able to be in this study. If you agree to be in this study and later decide to withdraw your participation, you may withdraw your authorization to use your PHI. This request must be made in writing to the Principal Investigator at kim.hammermsith@nationwidechildrens.org. If you withdraw your authorization, no new PHI may be collected and the PHI already collected may not be used unless it has already been used or is needed to complete the study analysis and reports. The results from this study may be published but your identity will not be revealed.

The PHI collected or created under this research study will be used or disclosed as needed until the end of the study. The records of this study will be kept for an indefinite period of time and your authorization to use or disclose your PHI will not expire.

10) WHOM SHOULD I CALL IF I HAVE QUESTIONS OR PROBLEMS?

If you have questions about anything while on this study or you have been injured by the research, you may contact the Principal Investigator at 614-722-1651 Monday - Friday. If you have questions, concerns, or complaints about the research; if you have questions about your rights as a research volunteer; if you cannot reach the Principal Investigator; or if you want to call someone else, call (614) 722-2708, Nationwide Children's Hospital Institutional Review Board, (the committee that reviews all research involving human subjects at Nationwide Children's Hospital).

The person completing this survey must be 18 years or older. If the individual with ectodermal dysplasia (ED) is under 18, their parent/guardian should complete this survey.

This survey may only be taken once. If you AND your child have ED, take the survey for yourself. If more than one child in the family has ED, take this survey for the OLDEST child.

This survey should take about 15 minutes to complete. By completing the survey below you are consenting to participate in this research study.

Are you:

◯ A person with ED
◯ A parent/guardian of a child with ED

PART 1/6

These first questions are about YOU, the person answering this survey.

Are you:

◯ Female
◯ Male
◯ Something else
◯ Rather not say

If something else, please explain:

____

What is your highest level of education?

◯ Some high school or less
◯ High school diploma or GED
◯ Associates or technical degree
◯ Bachelor's degree
◯ Graduate degree

What was your household income (USD) in 2023, before taxes?

◯ Under $25,000
◯ $25,000-49,999
◯ $50,000-74,999
◯ $75,000-99,999
◯ Over $100,000
◯ Rather not say

How many people in your immediate family (parents, siblings, spouse, and children) have ED?

____

PART 2/6

Thank you for telling us about yourself. The rest of the questions in this survey are about the person with ED. This may be you, or your child.

What year were you born?

____

What year was your child born?

____

What is your race/ethnicity? Select all that apply.

- ☐ American Indian or Alaska native
- ☐ Asian
- ☐ Black or African American
- ☐ Hispanic or Latino/a
- ☐ Middle Eastern
- ☐ Native Hawaiian or Pacific Islander
- ☐ White or Caucasian
- ☐ Other
- ☐ Rather not say

What is your child's race/ethnicity? Select all that apply.

- ☐ American Indian or Alaska native
- ☐ Asian
- ☐ Black or African American
- ☐ Hispanic or Latino/a
- ☐ Middle Eastern
- ☐ Native Hawaiian or Pacific Islander
- ☐ White or Caucasian
- ☐ Other
- ☐ Rather not say

If other, please explain:

____

Is your child:

- ◯ Female
- ◯ Male
- ◯ Something else
- ◯ Rather not say

If something else, please explain:

____

You live in a:

- ◯ Large city (>50,000 residents)
- ◯ Medium city (5,000-50,000 residents)
- ◯ Small town or city (< 5,000 residents)
- ◯ Don't know

Your child lives in a:

- ◯ Large city (>50,000 residents)
- ◯ Medium city (5,000-50,000 residents)
- ◯ Small town or city (< 5,000 residents)
- ◯ Don't know

PART 3/6

Next, we ask about the ED diagnosis.

What type of ED do you have?

- ◯ Hypohidrotic ectodermal dysplasia (HED)
- ◯ Hypohidrotic ectodermal dysplasia (HED) with immune deficiency
- ◯ Ectrodactyly ectodermal dysplasia and cleft lip/palate syndrome (EEC)
- ◯ Ankyloblepharon-ectodermal defects cleft lip/palate syndrome (AEC)
- ◯ Clouston syndrome/ectodermal dysplasia 2/hidrotic ectodermal dysplasia
- ◯ Focal dermal hypoplasia/Goltz syndrome/PORCN-related developmental disorder
- ◯ Incontinentia pigmenti (IP)
- ◯ Other
- ◯ Unknown

What type of ED does your child have?

- ◯ Hypohidrotic ectodermal dysplasia (HED)
- ◯ Hypohidrotic ectodermal dysplasia (HED) with immune deficiency
- ◯ Ectrodactyly ectodermal dysplasia and cleft lip/palate syndrome (EEC)
- ◯ Ankyloblepharon-ectodermal defects cleft lip/palate syndrome (AEC)
- ◯ Clouston syndrome/ectodermal dysplasia 2/hidrotic ectodermal dysplasia
- ◯ Focal dermal hypoplasia/Goltz syndrome/PORCN-related developmental disorder
- ◯ Incontinentia pigmenti (IP)
- ◯ Other
- ◯ Unknown

If other, please explain: ________________________________

About how long (after first getting medical help) did it take for you to get an ED diagnosis?

< 1 year ———————————— >10 years

(Place a mark on the scale above)

About how long (after first getting medical help) did it take for your child to get an ED diagnosis?

< 1 year ———————————— >10 years

(Place a mark on the scale above)

How many different providers have you seen to get a diagnosis? ________________________

How many different providers has your child seen to get a diagnosis? ________________________

Who was the first person to suggest that you had ED?

- ◯ Primary care doctor
- ◯ Medical specialist
- ◯ Dentist
- ◯ Other

Who was the first person to suggest that your child had ED?	◯ Primary care doctor<br>◯ Medical specialist<br>◯ Dentist<br>◯ Other
Please describe:	_______________________________________
At the time of diagnosis, were you given enough information on your condition?	◯ Yes<br>◯ No<br>◯ Not applicable
At the time of diagnosis, were you given enough information on your child's condition?	◯ Yes<br>◯ No<br>◯ Not applicable
What information were you missing?	_______________________________________
How was your experience getting care after being diagnosed with ED?	◯ Easier to get care<br>◯ No change<br>◯ Harder to get care
Has your ED diagnosis been confirmed with genetic testing?	◯ Yes<br>◯ No<br>◯ Not applicable
Has your child's ED diagnosis been confirmed with genetic testing?	◯ Yes<br>◯ No<br>◯ Not applicable
If no, why not?	◯ Testing in progress or plan to complete<br>◯ Testing completed but was inconclusive<br>◯ Cost of testing is too high<br>◯ Not interested in testing<br>◯ Other reason
Please describe:	_______________________________________

PART 4/6

Thank you. Next are questions about insurance.

What kind of medical insurance do you have? Select all that apply.

- ☐ Private insurance through an employer
- ☐ Private insurance purchased on my own
- ☐ Medicare
- ☐ Medicaid
- ☐ Military healthcare (VA, TriCare, CHAMP-VA)
- ☐ Other
- ☐ None
- ☐ Not sure

What kind of medical insurance does your child with ED have? Select all that apply.

- ☐ Private insurance through an employer
- ☐ Private insurance purchased on my own
- ☐ Medicare
- ☐ Medicaid
- ☐ Military healthcare (VA, TriCare, CHAMP-VA)
- ☐ Other
- ☐ None
- ☐ Not sure

If other, please explain: ______________________

What kind of dental insurance do you have?

- ◯ Private dental insurance
- ◯ Public dental insurance (Medicaid or CHIP)
- ◯ No dental insurance
- ◯ Not sure

What kind of dental insurance does your child have?

- ◯ Private dental insurance
- ◯ Public dental insurance (Medicaid or CHIP)
- ◯ No dental insurance
- ◯ Not sure

About how much money did you pay for medical expenses (physician visit co-pays, deductibles, co-insurance, prescriptions) for yourself, in the year 2023?

- ◯ $0-$499
- ◯ $500-$999
- ◯ $1,000-$1,499
- ◯ $1,500-$1,999
- ◯ $2,000-$2,999
- ◯ Over $3,000
- ◯ Don't know

About how much money did you pay for medical expenses (physician visit co-pays, deductibles, co-insurance, prescriptions) for your child, in the year 2023?

- ◯ $0-$499
- ◯ $500-$999
- ◯ $1,000-$1,499
- ◯ $1,500-$1,999
- ◯ $2,000-$2,999
- ◯ Over $3,000
- ◯ Don't know

About how much money have you paid for your dental expenses in your lifetime?

Less than $1,000 — More than $60,000

(Place a mark on the scale above)

About how much money have you paid for dental expenses for your child, in their lifetime?

Less than $1,000 — More than $60,000

(Place a mark on the scale above)

Have you been denied any of these because of insurance? Select all that apply.	☐ Diagnostic test<br>☐ Medication that is FDA approved or commercially available for your/your family child's condition<br>☐ Investigational treatment (e.g., medication that is NOT FDA approved or commercially available)<br>☐ Device or medical equipment<br>☐ Medical service (e.g., occupational therapy)<br>☐ Referral to a medical or dental specialist<br>☐ Other
Has your child been denied any of these because of insurance? Select all that apply.	☐ Diagnostic test<br>☐ Medication that is FDA approved or commercially available for your/your family child's condition<br>☐ Investigational treatment (e.g., medication that is NOT FDA approved or commercially available)<br>☐ Device or medical equipment<br>☐ Medical service (e.g., occupational therapy)<br>☐ Referral to a medical or dental specialist<br>☐ Other
If other, please explain	__________________________________
In the past 12 months, did you receive counseling or therapy from a mental health professional such as a psychiatrist, psychologist, psychiatric nurse, or clinical social worker?	◯ Yes<br>◯ No<br>◯ Unsure
In the past 12 months, did your child receive counseling or therapy from a mental health professional such as a psychiatrist, psychologist, psychiatric nurse, or clinical social worker?	◯ Yes<br>◯ No<br>◯ Unsure

PART 5/6
Thank you. Next are questions about dental care.

Have you seen a dentist in the past 12 months?

- ◯ Yes
- ◯ No
- ◯ Unsure

Has your child seen a dentist in the past 12 months?

- ◯ Yes
- ◯ No
- ◯ Unsure

Which dental specialists have you seen? Select all that apply.

- ☐ General/family dentist
- ☐ Pediatric dentist
- ☐ Orthodontist
- ☐ Prosthodontist
- ☐ Periodontist
- ☐ Oral surgeon
- ☐ Other

Which dental specialists has your child seen? Select all that apply.

- ☐ General/family dentist
- ☐ Pediatric dentist
- ☐ Orthodontist
- ☐ Prosthodontist
- ☐ Periodontist
- ☐ Oral surgeon
- ☐ Other

If other, please explain:

____

Overall, how would you rate the health of your teeth and gums?

- ◯ Excellent
- ◯ Very good
- ◯ Good
- ◯ Fair
- ◯ Poor

Overall, how would you rate the health of your child's teeth and gums?

- ◯ Excellent
- ◯ Very good
- ◯ Good
- ◯ Fair
- ◯ Poor

In the last year, how often have you felt life in general was less satisfying because of problems with your teeth, mouth, or dentures?

- ◯ Very often
- ◯ Fairly often
- ◯ Occasionally
- ◯ Hardly ever
- ◯ Never

In the last year, how often has your child felt life in general was less satisfying because of problems with their teeth, mouth, or dentures?

- ◯ Very often
- ◯ Fairly often
- ◯ Occasionally
- ◯ Hardly ever
- ◯ Never

In the last year, how often have you had difficulty doing usual jobs or attending school because of problems with your teeth, mouth or dentures?	◯ Very often<br>◯ Fairly often<br>◯ Occasionally<br>◯ Hardly ever<br>◯ Never
In the last year, how often has your child had difficulty doing usual jobs or attending school because of problems with their teeth, mouth or dentures?	◯ Very often<br>◯ Fairly often<br>◯ Occasionally<br>◯ Hardly ever<br>◯ Never
In the last year, how often have you avoided particular foods because of problems with your teeth, mouth, or dentures?	◯ Very often<br>◯ Fairly often<br>◯ Occasionally<br>◯ Hardly ever<br>◯ Never
In the last year, how often has your child avoided particular foods because of problems with their teeth, mouth, or dentures?	◯ Very often<br>◯ Fairly often<br>◯ Occasionally<br>◯ Hardly ever<br>◯ Never
In the last year, how often have you been self-conscious or embarrassed because of your teeth, mouth, or dentures?	◯ Very often<br>◯ Fairly often<br>◯ Occasionally<br>◯ Hardly ever<br>◯ Never
In the last year, how often has your child been self-conscious or embarrassed because of their teeth, mouth, or dentures?	◯ Very often<br>◯ Fairly often<br>◯ Occasionally<br>◯ Hardly ever<br>◯ Never

Please think of the first DENTAL provider you saw when you starting having problems with your ED.

How would you rate their...

	Poor	Fair	Neutral	Good	Excellent	Not applicable
Knowledge of ED?	○	○	○	○	○	○
Willingness to ask other dentists/medical colleagues for help?	○	○	○	○	○	○
Willingness to research ED themselves?	○	○	○	○	○	○

Please think of the first DENTAL provider your child saw when they starting having problems with their ED.

How would you rate their...

	Poor	Fair	Neutral	Good	Excellent	Not applicable
Knowledge of ED?	◯	◯	◯	◯	◯	◯
Willingness to ask other dentists/medical colleagues for help?	◯	◯	◯	◯	◯	◯
Willingness to research ED themselves?	◯	◯	◯	◯	◯	◯

Please think of the first MEDICAL provider you saw when you starting having problems with your ED.

How would you rate their...

	Poor	Fair	Neutral	Good	Excellent	Not applicable
Knowledge of ED?	◯	◯	◯	◯	◯	◯
Willingness to ask other dentists/medical colleagues for help?	◯	◯	◯	◯	◯	◯
Willingness to research ED themselves?	◯	◯	◯	◯	◯	◯

Please think of the first MEDICAL provider your child saw when they starting having problems with their ED.

How would you rate their...

	Poor	Fair	Neutral	Good	Excellent	Not applicable
Knowledge of ED?	◯	◯	◯	◯	◯	◯
Willingness to ask other dentists/medical colleagues for help?	◯	◯	◯	◯	◯	◯
Willingness to research ED themselves?	◯	◯	◯	◯	◯	◯

PART 6/6

Last, we ask about challenges in getting care.

In a normal year, how many different medical and dental specialists do you see for your ED? ______________________

In a normal year, how many different medical and dental specialists does your child see for their ED? ______________________

How far do you usually travel (in miles) to get medical care?
0 150 300+
(Place a mark on the scale above)

How far do you usually travel (in miles) to get medical care for your child?
0 150 300+
(Place a mark on the scale above)

What is the most distance you have ever traveled (in miles) to get medical care?
0 150 300+
(Place a mark on the scale above)

What is the most distance you have ever traveled (in miles) to get medical care for your child?
0 150 300+
(Place a mark on the scale above)

How far do you usually travel (in miles) to get dental care?
0 150 300+
(Place a mark on the scale above)

How far do you usually travel (in miles) to get dental care for your child?
0 150 300+
(Place a mark on the scale above)

What is the most distance you have ever traveled (in miles) to get dental care?
0 150 300+
(Place a mark on the scale above)

What is the most distance you have ever traveled (in miles) to get dental care for your child?
0 150 300+
(Place a mark on the scale above)

Have you needed to relocate in order to access treatment or clinical trials for ED long-term?
- ◯ Yes, relocated in-state
- ◯ Yes, relocated out-of-state
- ◯ No

Has your family needed to relocate in order to access treatment or clinical trials for ED long-term?
- ◯ Yes, relocated in-state
- ◯ Yes, relocated out-of-state
- ◯ No

If you relocated, please describe:

____

Do you agree or disagree that you have received sufficient support in the following areas?

	Strongly disagree	Disagree	Neutral (neither agree nor disagree)	Agree	Strongly agree	Don't know	Not applicable
Medical (i.e. doctors, nurses)	○	○	○	○	○	○	○
Dental	○	○	○	○	○	○	○
Social (i.e. family, friends, church members)	○	○	○	○	○	○	○
Financial (i.e. insurance coverage)	○	○	○	○	○	○	○
Psychological (i.e. mental health care, counselors)	○	○	○	○	○	○	○

Do you agree or disagree that your child has received sufficient support in the following areas?

	Strongly disagree	Disagree	Neutral (neither agree nor disagree)	Agree	Strongly agree	Don't know	Not applicable
Medical (i.e. doctors, nurses)	○	○	○	○	○	○	○
Dental	○	○	○	○	○	○	○
Social (i.e. family, friends, church members)	○	○	○	○	○	○	○
Financial (i.e. insurance coverage)	○	○	○	○	○	○	○
Psychological (i.e. mental health care, counselors)	○	○	○	○	○	○	○

Did any of the following limit your ability to get MEDICAL care for ED?

	Never	Sometimes	About half the time	Most of the time	Almost always	Not applicable
Finances	○	○	○	○	○	○
Travel distance	○	○	○	○	○	○
Difficulty getting time off of school or work	○	○	○	○	○	○
Lack of childcare	○	○	○	○	○	○
Lack of or delay in referrals	○	○	○	○	○	○
Challenges with insurance coverage	○	○	○	○	○	○

Did any of the following limit your child's ability to get MEDICAL care for ED?

	Never	Sometimes	About half the time	Most of the time	Almost always	Not applicable
Finances	◯	◯	◯	◯	◯	◯
Travel distance	◯	◯	◯	◯	◯	◯
Difficulty getting time off of school or work	◯	◯	◯	◯	◯	◯
Lack of childcare	◯	◯	◯	◯	◯	◯
Lack of or delay in referrals	◯	◯	◯	◯	◯	◯
Challenges with insurance coverage	◯	◯	◯	◯	◯	◯

Did any of the following limit your ability to get DENTAL care for ED?

	Never	Sometimes	About half the time	Most of the time	Almost always	Not applicable
Finances	○	○	○	○	○	○
Travel distance	○	○	○	○	○	○
Difficulty getting time off of school or work	○	○	○	○	○	○
Lack of childcare	○	○	○	○	○	○
Lack of or delay in referrals	○	○	○	○	○	○
Challenges with insurance coverage	○	○	○	○	○	○

Did any of the following limit your child's ability to get DENTAL care for ED?

	Never	Sometimes	About half the time	Most of the time	Almost always	Not applicable
Finances	◯	◯	◯	◯	◯	◯
Travel distance	◯	◯	◯	◯	◯	◯
Difficulty getting time off of school or work	◯	◯	◯	◯	◯	◯
Lack of childcare	◯	◯	◯	◯	◯	◯
Lack of or delay in referrals	◯	◯	◯	◯	◯	◯
Challenges with insurance coverage	◯	◯	◯	◯	◯	◯

This is the final page of the survey.

How strongly do you agree or disagree with the following statements? Think about your overall experience with healthcare when responding. Note: "healthcare providers" includes medical, dental, and other types of providers you may have seen.

	Strongly disagree	Disagree	Neutral (neither agree nor disagree)	Agree	Strongly agree	Don't know	Not applicable
Healthcare providers are good about explaining the reason for medical tests	○	○	○	○	○	○	○
I think my healthcare providers' office has everything needed to provide complete medical care	○	○	○	○	○	○	○
The medical care I have been receiving is just about perfect	○	○	○	○	○	○	○
Sometimes healthcare providers make me wonder if their diagnosis is correct	○	○	○	○	○	○	○
I feel confident that I can get the medical care I need without being set back financially	○	○	○	○	○	○	○
When I go for medical care, they are careful to check everything when treating and examining me	○	○	○	○	○	○	○
I have to pay for more of my medical care than I can afford	○	○	○	○	○	○	○
I have easy access to the medical specialists I need	○	○	○	○	○	○	○
Where I get medical care, I have to wait too long for emergency treatment	○	○	○	○	○	○	○
Healthcare providers act too businesslike and impersonal toward me	○	○	○	○	○	○	○
My healthcare providers treat me in a very friendly and courteous manner	○	○	○	○	○	○	○
Those who provide me medical care sometimes hurry too much when they treat me	○	○	○	○	○	○	○

Healthcare providers sometimes ignore what I tell them	○	○	○	○	○	○	○
I have some doubts about the ability of the healthcare providers who treat me	○	○	○	○	○	○	○
Healthcare providers usually spend plenty of time with me	○	○	○	○	○	○	○
I find it hard to get an appointment for medical care right away	○	○	○	○	○	○	○
I am dissatisfied with some things about the medical care I receive	○	○	○	○	○	○	○
I am able to get medical care whenever I need it	○	○	○	○	○	○	○

This is the final page of the survey.

How strongly do you agree or disagree with the following statements? Think about your child's overall experience with healthcare when responding. Note: "healthcare providers" includes medical, dental, and other types of providers you may have seen.

Please answer these questions in terms of how YOUR CHILD is feeling. If your child is under age 10, please answer based on your experience with them as a caregiver.

	Strongly disagree	Disagree	Neutral (neither agree nor disagree)	Agree	Strongly agree	Don't know	Not applicable
Healthcare providers are good about explaining the reason for medical tests	○	○	○	○	○	○	○
I think my healthcare providers' office has everything needed to provide complete medical care	○	○	○	○	○	○	○
The medical care I have been receiving is just about perfect	○	○	○	○	○	○	○
Sometimes healthcare providers make me wonder if their diagnosis is correct	○	○	○	○	○	○	○
I feel confident that I can get the medical care I need without being set back financially	○	○	○	○	○	○	○
When I go for medical care, they are careful to check everything when treating and examining me	○	○	○	○	○	○	○
I have to pay for more of my medical care than I can afford	○	○	○	○	○	○	○
I have easy access to the medical specialists I need	○	○	○	○	○	○	○
Where I get medical care, I have to wait too long for emergency treatment	○	○	○	○	○	○	○
Healthcare providers act too businesslike and impersonal toward me	○	○	○	○	○	○	○

My healthcare providers treat me in a very friendly and courteous manner	○	○	○	○	○	○	○
Those who provide me medical care sometimes hurry too much when they treat me	○	○	○	○	○	○	○
Healthcare providers sometimes ignore what I tell them	○	○	○	○	○	○	○
I have some doubts about the ability of the healthcare providers who treat me	○	○	○	○	○	○	○
Healthcare providers usually spend plenty of time with me	○	○	○	○	○	○	○
I find it hard to get an appointment for medical care right away	○	○	○	○	○	○	○
I am dissatisfied with some things about the medical care I receive	○	○	○	○	○	○	○
I am able to get medical care whenever I need it	○	○	○	○	○	○	○

This is the end. Thank you very much for your time and participation!

Are you interested in a one-hour focus group to help researchers learn more about your experience with ED? Focus groups will be held online, at a time that works for you.	◯ Yes<br>◯ No
