# Supplementary material for: Assessing healthcare experiences and barriers to care among individuals with ectodermal dysplasia
Source: Orphanet J Rare Dis. 2026 Mar 5;21:147. doi: 10.1186/s13023-026-04247-z (PMC13072512; doi:10.1186/s13023-026-04247-z)
Supplement: Supplementary file 2 — Supplementary Material 2 [file 13023_2026_4247_MOESM2_ESM.pdf]

Introduction

Good morning/afternoon/evening. Thank you so much for agreeing to participate in this study. My name is Amanda Swanson and I will be leading the focus group today. I am a pediatric dental resident at Nationwide Children's Hospital in Columbus, Ohio. Our discussion today will help our team learn more about the results of the survey that you have already done. Specifically, we want to learn about your attitudes, beliefs, and experiences related to getting medical and dental care for [you/your child]. Our conversation is expected to last about an hour.

Objectives of Focus Group

We want to learn more about challenges faced by patients with ectodermal dysplasia and their families when accessing healthcare and health services. This will allow us to identify areas of need for advocacy, research, patient education, and support from the NFED.

This focus group will help us to:

- Contextualize the challenges faced by patients with ED when obtaining a diagnosis and seeking care
- Explore how the attitudes and experiences of families affected by ectodermal dysplasia differ from the broader rare disease community
- Evaluate barriers and facilitators of effective care
- Assess presence and extent of differences in experiences with medical vs dental care
- Identify areas for improvement at the point-of-care, community, and public policy levels

Confidentiality and Anonymity

This focus group will be recorded on an audio device to allow for transcription and analysis to achieve the research objectives. Your comments and the recording will remain strictly anonymous. Any names or personal identifiers that you share will be removed to maintain confidentiality.

Focus Group Guide

Subject	Questions
**Diagnosis**	• Tell me about your experience being diagnosed with ectodermal dysplasia.<br>• What was the most challenging part about obtaining a diagnosis? How did going through this challenge impact you?<br>• Can you speak about the process of obtaining healthcare services and supports following your diagnosis? Was there a change in your ability to access them?
**Provider Satisfaction**	• Please describe your overall experiences and satisfaction with healthcare providers you/your family member visited since the time of diagnosis.<br>• Think of one healthcare provider with whom you have worked that has had a particularly positive impact on your care.<br>○ Describe this individual and your interactions with them.<br>○ What made this experience effective? What could other providers do to match this?<br>• Think of one healthcare provider with whom you have worked that has had a particularly negative or neutral impact on your care.<br>○ Describe this individual and your interactions with them.<br>○ What made this experience ineffective? What could this provider have done to make your experience better?
**Oral Health**	• Tell me about your experiences with dentistry and dental providers.<br>○ What has been most helpful for you/your family in the process of seeking dental care and accessing oral health services?<br>○ What has been most challenging for you/your family in the process of seeking dental care and accessing oral health services?<br>• How do you value oral health? Said another way, where would you place oral health in a priority list of other health topics and why?<br>• Describe an oral health success story (if applicable).
**Access to Care**	• What has been most helpful for you/your family in the process of seeking care and accessing services?<br>• What has been most challenging for you/your family in the process of seeking care and accessing services?<br>• How has medical insurance helped or hindered your ability to access care? What about dental insurance?
**Conclusion**	• What have your interactions been like with the NFED and/or others in the ectodermal dysplasia community?<br>• Are there additional supports that have benefitted your family in terms of navigating the healthcare system?<br>• If there were ONE SINGLE THING that could be provided by the healthcare system to improve your/your family's experience with ectodermal dysplasia, what would it be?<br>• Is there anything that you would like to add about your experience with ectodermal dysplasia that will help us better understand the challenges experienced by patients/families?
